# Characterization of interprofessional education experiences in health education at the University of São Paulo

**DOI:** 10.1590/1980-220X-REEUSP-2023-0118en

**Published:** 2023-11-24

**Authors:** Jaqueline Bissolati Costa, Nathalia Romeu de Mazzi, Marina Peduzzi, Ana Claudia Camargo Gonçalves Germani, Jaqueline Alcântara Marcelino Silva, Heloise Lima Fernandes Agreli, Ariana Gomes Nascimento, Gisele Silvestre Belber, Valéria Marli Leonello

**Affiliations:** 1 Universidade de São Paulo, São Paulo, SP, Brasil.; 2 Universidade Federal de São Carlos, São Carlos, SP, Brasil.

**Keywords:** Teaching, Interprofessional Education, Surveys and Questionnaires, Enseñanza, Educación Interprofesional, Encuestas y Cuestionarios, Ensino, Educação Interprofissional, Inquéritos e Questionários

## Abstract

**Objective:**

To map the experiences of Interprofessional Education (IPE) in Health at the São Paulo campus of the University of São Paulo.

**Method:**

This is a descriptive, exploratory study with a quantitative approach and data collection through an online questionnaire addressed to teachers from eight teaching units and 14 health courses. The data was analyzed using absolute and percentage frequencies.

**Results:**

The majority of teachers do not take part in IPE experiences (70.4%). Most of the experiences are between six and ten years old, involving up to four teachers and small groups of students, mostly extracurricular or extension activities. The teaching and assessment strategies are diverse and open to students of all years.

**Conclusion:**

IPE experiences involve a small number of students and teachers and, although consolidated in terms of the time they have been offered, are limited to extracurricular spaces. Factors such as improvements in institutional support and teacher development are pointed out as important for making progress in strengthening IPE in the analyzed context.

## RESUMO

**Objetivo:**

Mapear as experiências de Educação Interprofissional (EIP) em Saúde no campus São Paulo da Universidade de São Paulo.

**Método:**

Pesquisa descritiva, exploratória, com abordagem quantitativa e coleta de dados por meio de um questionário online, direcionado aos docentes de oito unidades de ensino e 14 cursos da área de saúde. Os dados foram analisados por meio de frequências absolutas e percentuais.

**Resultados:**

A maior parte dos docentes não participa de experiências de EIP (70,4%). Em sua maioria, as experiências têm de seis a dez anos de existência, com envolvimento de até quatro docentes e em pequenos grupos de estudantes, majoritariamente extracurriculares ou atividades de extensão. As estratégias de ensino e avaliação são diversificadas e abertas aos estudantes de todos os anos.

**Conclusão:**

As experiências de EIP envolvem um número pequeno de estudantes e professores e, embora consolidadas em relação ao tempo de oferta, são limitadas aos espaços extracurriculares. Fatores como melhorias no apoio institucional e desenvolvimento docente são apontados como importantes para avançar no fortalecimento da EIP no contexto analisado.

## RESUMEN

**Objetivo:**

Mapear las experiencias de Educación Interprofesional (EIP) en Salud en el campus de São Paulo de la Universidad de São Paulo.

**Método:**

Se trata de un estudio descriptivo, exploratorio, con abordaje cuantitativo y recolección de datos por medio de cuestionario online dirigido a profesores de ocho unidades de enseñanza y 14 cursos de salud. Los datos se analizaron mediante frecuencias absolutas y porcentuales.

**Resultados:**

La mayoría de los profesores no participa en experiencias de EIP (70,4%). La mayoría de las experiencias tienen entre seis y diez años de antigüedad, involucran hasta cuatro profesores y pequeños grupos de estudiantes, en su mayoría actividades extracurriculares o de extensión. Las estrategias de enseñanza y evaluación son diversas y están abiertas a estudiantes de todos los cursos.

**Conclusión:**

Las experiencias de EIP involucran a un número reducido de estudiantes y profesores y, aunque consolidadas en términos de oferta de tiempo, se limitan a espacios extracurriculares. Factores como la mejora del apoyo institucional y el desarrollo de las docentes se señalan como importantes para avanzar en el fortalecimiento de la EIP en el contexto analizado.

## INTRODUCTION

Health professions’ education is traditionally and mostly characterized by the uniprofessional model, in which students from one professional area learn skills specific to their profession, with little or limited contact and learning with students from other professional areas. This training model has reinforced the fragmentation and piecemeal nature of health care, impacting on the safety and quality of care offered to users, families and communities^(1)^. On the other hand, the change in the demographic and epidemiological profile reiterates the importance of integrated health care, with the need for teamwork, articulated actions between teams, services and the health care network, which requires professional training from an interprofessional perspective^(2)^.

In this sense, Interprofessional Education in health (IPE) appears to be a necessary teaching approach, understood as being articulated with professional practices. IPE occurs when at least two students from different training areas have the opportunity to learn in a shared way, providing an exchange of knowledge and experiences, clarification of roles and effective communication, with the aim of collaborative work^(3)^. Different authors have reported in the literature that IPE experiences have a positive impact on structuring interprofessional work and improving the quality of care provided to users of health systems, bringing greater satisfaction to both those who provide care and those who receive it^(4)^.

It is therefore essential to invest in initiatives that strengthen interprofessional education practices and their inclusion in higher education training models. To this end, we should consider the different factors that contribute to its promotion, such as structural determinants related to education and health policies, which support and sustain both interprofessional collaboration and interprofessional education; organizational determinants, related to institutional support and teacher training; and interactional determinants, which refer to the interactions and behaviors of the professionals encompassed by the team^(5)^. In Brazil, there is a correlation between the elements of interprofessional work and the guiding principles of the Unified Health System (SUS), especially comprehensiveness, understood as an articulated set of individual and collective preventive and curative health actions and services. Therefore, there must be coordination between the various health professionals and the services offered, in order to provide health care to the population^(3)^. In the same vein, the guidelines that orient the training of professionals have been recently undergoing changes towards greater integration.

The National Curriculum Guidelines (DCN in the Portuguese acronym) for undergraduate health courses^(6)^ emphasize teamwork as a necessary skill for health work^(7,8)^. In 2017, the National Health Council approved Resolution 569, which establishes common assumptions, principles and guidelines for the DCN of all undergraduate health courses, reaffirming the defense of the SUS, meeting social needs, teaching-­service-management-community integration and comprehensive care, considering interprofessional work and education, communication and teaching methodologies that provide collaborative and meaningful learning as guiding principles for the profile of graduates^(9)^. However, higher education for health professionals using this logic is still in its infancy, with the main barriers being physical spaces, with courses separated by location or campus, teaching staff unprepared for interprofessional education and the predominantly uniprofessional training model^(7)^.

Brazilian experiences of interprofessional education in the health area are recent and generally limited to extracurricular activities, such as extension projects and elective courses^(10)^. Experiences are generally happening in isolation and are often limited to a few courses and initiatives by faculty involved in interprofessional education, as well as inductive policies such as the Education through Work Program (PET-Saúde)^(11)^. Strengthening these initiatives and more effective policies for implementing interprofessional education in the curricula of undergraduate health courses needs to be based on a diagnosis of the structural scenario of interprofessional education in the various institutions. By mapping the IPE experiences offered to undergraduate students, it is possible to measure the potential and challenges to be faced by the institution in order to widely implement these practices in their training curricula.

Therefore, this study was aimed to answer the following question: *What are the IPE experiences in the units participating in the Work Education Program for Health/Interprofessionality (PET-Saúde/Interprofissionalidade) on the São Paulo campus of the University of São Paulo (USP*)? This study assumes IPE as its object, in terms of mapping the experiences developed on USP’s São Paulo campus, in the units participating in PET-Saúde/Interprofissionalidade. Data collection based on the perceptions and participation of teachers was chosen for a better diagnosis of how the experiences are being implemented in the institution and based on the principles of IPE for social action in the provision of comprehensive quality healthcare.

## METHOD

### Study Design

This is a descriptive, exploratory and cross-sectional study, with a quantitative approach and data collection through interviews, using a structured questionnaire that enabled a broad mapping for the description of IPE experiences of USP teachers.

### Location of the Study

The study was carried out on USP’s São Paulo campus, in eight teaching units: the School of Sciences, Arts and Humanities, the School of Physical Education and Sport, the School of Nursing, the School of Pharmaceutical Sciences, the School of Medicine, the School of Dentistry, the School of Public Health and the Institute of Psychology. The survey involved 14 undergraduate courses: Physical Education and Health, Physical Education and Sports, Nursing, Pharmacy, Physiotherapy, Speech Therapy, Gerontology, Medicine, Nutrition, Obstetrics, Dentistry, Psychology, Public Health and Occupational Therapy.

### Population

The population encompassed teachers from undergraduate health courses at the units participating in PET-Saúde/Interprofissionalidade on USP’s São Paulo campus. According to the institution’s statistical yearbook, the total population of permanent teachers in the units surveyed was 1,015 in 2021. It should be noted that the information obtained in the yearbook is overestimated in one of the units analyzed, as it was not possible to find the information on the number of teachers disaggregated by course, being counted by unit instead^(12)^.

### Selection Criteria

The inclusion criteria were: teachers linked to the participating units involved in undergraduate activities, from the three work regimes: part-time (RTP in the Portuguese acronym); full time (RTC in the Portuguese acronym); and full-time teaching and research (RDIDP in the Portuguese acronym)^(13)^. The exclusion criteria were: teachers on leave, on any kind of leave and who were not involved in activities related to undergraduate teaching during the data collection period.

### Data Collection

Data collection took place between October 15, 2020 and May 25, 2021. An electronic questionnaire was applied containing 33 questions subdivided into four blocks: undergraduate health course, IPE initiatives, respondent profile and participation in IPE. The questionnaire was based on a study^(14)^ that mapped IPE experiences in Brazil. To apply the questionnaire, a pilot test was carried out with eight health professors who were not part of the sample, in order to improve the questions in the context analyzed.

**Figure 1:**
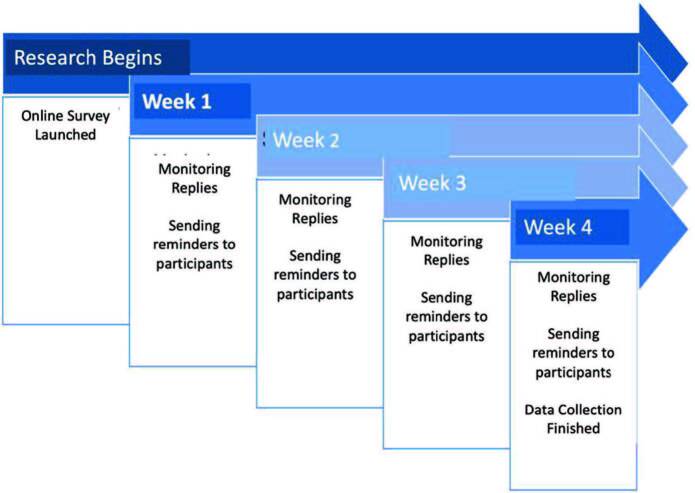
– Flowchart of data collection, based on research procedures. University of São Paulo (USP) – São Paulo, Brazil, 2022.

For data collection, an online questionnaire was used, using the Survey Monkey® platform. Considering that the data was collected during the COVID-19 pandemic, the methodology was suited to the context of social isolation and university closures, with activities being relocated to a remote format. Although the main limitation of the survey method is the low response rate, estimated at 10% to 15% of responses to the invitations sent out^(15)^, an attempt was made to mitigate this effect by sending reminders by email so that those invited could respond to the questionnaire. These reminders were sent out weekly for four weeks after the invitation and the link to access the survey form were sent out, as detailed in Figure 1.

The data collection cycles always began on Mondays and ended on Sundays. E-mail invitations were sent out to teachers by teaching unit, with a total of seven consecutive days per cycle, with four collection cycles per teaching unit, considering the first week of sending the invitation and three weeks of monitoring and reminders, totaling a collection period of nine months.

### Data Analysis and Processing

The data was extracted from the Survey Monkey® platform into a database using the Microsoft Excel® program and then imported into the Statistical Package for the Social Sciences (SPSS), version 20.05. Descriptive statistics were used for the analysis, using absolute and relative frequencies. 

### Ethical Aspects

This research was cleared by the Research Ethics Committee of the USP School of Nursing, under opinion no. 3.647.173, in 2020, and was carried out in accordance with the recommendations of Resolution no. 466/2012 of the National Health Council^(16)^. The teachers were invited to participate and were informed about the objectives, the research method and the guarantee of confidentiality and anonymity. The Informed Consent Form (ICF) was presented to all participants in the research in an online format. Participants were instructed to read the ICF and express their agreement or otherwise to the question: “Have you understood the guidelines and do you agree to participate freely, knowledgeably and spontaneously in this research?”. If they agreed, they were asked to enter their full name in full so that it could be attached to their acceptance to take part in the study. The participant was then directed to the beginning of the questionnaire. If not, the participant received a thank you note and the contact was closed.

## RESULTS

A total of 125 teachers took part in the survey. Of this sample, 70.4% (n = 88) answered that they had not taken part in IPE experiences. Among the teachers, 29.6% (n = 37) said they had taken part in IPE experiences, of which 81.1% (n = 30) shared their experiences, answering the specific questions on the subject. The distribution of experiences was as follows: 70.3% (n = 26) said they had only taken part in one experience, while 10.8% (n = 4) shared two experiences. This question had an abstention rate of 18.9% (n = 7).

The results on the characteristics of the IPE experiences in the study sample are shown in Table 1.

**Table 1–:** Distribution of relative frequencies and percentages for the answers obtained in the characterization of IPE experiences at USP – São Paulo, Brazil, 2022.

DESCRIPTOR	NABSOLUTE (%)
**Number of teachers – classes**	N total = 34
One	5 (14.7)
Two	6 (17.6)
Three to four	11 (32.4)
Five to six	4 (11.8)
More than six	8 (23.5)
**Number of students involved**	N total = 33
Up to 20	10 (30.3)
21 – 40	7 (21.2)
41 – 60	7 (21.2)
61 – 80	3 (9.1)
81 or more	6 (18.2)
No data	1 (3)
**Course of the students involved** ^1^	N total = 34
Psychology	17 (50.0)
Medicine	16 (47.1)
Physiotherapy	15 (44.1)
Nursing	15 (44.1)
Nutrition	14 (41.2)
Public Health	13 (38.2)
Pharmacy	12 (35.3)
Occupational therapy	12 (35.3)
Speech therapy	11 (32.4)
Dentistry	10 (29.4)
Gerontology	9 (26.5)
Obstetrics	9 (26.5)
Physical education and health	8 (23.5)
Physical education and sports	7 (20.6)
Others	15 (44.1)
**Course of the teachers involved** ^1^	N total = 34
Nursing	11 (32.4)
Nutrition	11 (32.4)
Medicine	11 (32.4)
Public Health	10 (29.4)
Psychology	10 (29.4)
Pharmacy	9 (26.5)
Physiotherapy	8 (23.5)
Dentistry	8 (23.5)
Occupational Therapy	7 (20.6)
Physical education and health	5 (14.7)
Speech therapy	5 (14.7)
Gerontology	5 (14.7)
Obstetrics	4 (11.8)
Physical education and sports	4 (11.8)
Outros	4 (11.8)
**Teaching/learning strategy adopted** ^1^	N total = 34
Group discussion	24 (70.6)
Dialogic lecture	21 (61.8)
Text discussion	16 (47.1)
Seminar	15 (44.1)
Case study	14 (41.2)
Practical class	12 (35.3)
Problematization	12 (35.3)
Project-based learning	11 (32.4)
Team-Based Learning	10 (29.4)
Problem-Based Learning	10 (29.4)
Lecture	9 (26.5)
Simulation	7 (20.6)
Other	7 (20.6)
**Teaching/learning assessment strategy adopted** ^1^	N total = 24
Collective production	10 (41.7)
Field diary	9 (37.5)
Seminar	8 (33.3)
Written test	3 (12.5)
Portfolio	1 (4.2)
Simulation	1 (4.2)
Other	10 (41.7)
No data	10 (41.7)
**Strategy used for the student to evaluate the experience** ^1^	N total = 34
Meetings/conversations with students	23 (67.6)
Specific instrument with questions	11 (32.4)
No evaluation	4 (11.8)
Other	9 (26.5)
**Time (years) that the IPE initiative has been running – classes**	N total = 32
1 to 5 years	10 (32.3)
6 to 10 years	14 (45.2)
11 years or more	7 (22.6)
No data	3 (9.3)
**Moment in which IPE takes place**	N total = 34
Open to all stages of the student’s education	13 (38.2)
At the end of the course	4 (11.8)
At the beginning of the course	1 (2.9)
During the course	16 (47.1)
**The initiative is linked to:** ^1^	N total = 34
To Teaching (non-compulsory subject/axis/module)	17 (50.0)
To Extension/Community Reach-out	16 (47.1)
To Teaching (compulsory subject/axis/module)	12 (35.3)
**Venue of the initiative** ^1^	N total = 34
Classroom	19 (55.9)
Community/territory	12 (35.3)
School	9 (26.5)
Basic Health Unit/Family Health Unit	7 (20.6)
Laboratory	7 (20.6)
Hospital	5 (14.7)
Other	7 (20.6)
**This initiative is provided for in the Course’s Pedagogical Project (PPC)**	N total = 34
Yes	22 (64.7)
No	8 (23.5)
I don’t know	4 (11.8)
**Does the teacher coordinate the initiative?**	N total = 34
No	3 (8.8)
Yes	31 (91.2)

Note: ^1^Multiple answer – the sum of the percentages does not add up to 100%. The sum of the percentages may not add up to 100% due to rounding. The sample consisted of 37 teachers who said they participated in initiatives on the subject, but only 30 described the experiences they had implemented.

We found that 32.4% of IPE experiences involve the participation of three to four teachers and 23.5% involve six or more teachers. In addition, 30.3% involve up to 20 students. It should also be noted that 45.2% of these experiences have been taking place at the institution for between six and ten years, indicating that the experiences are already consolidated (Table 1).

With regard to courses, more than 40% of IPEs involve students from Psychology (50.0%), Medicine (47.1%), Physiotherapy (44.1%), Nursing (44.1%) and Nutrition (41.2%). With regard to teachers, with equal frequencies of 32.4%, the courses with the greatest participation of professionals were Nursing, Nutrition and Medicine, followed by teachers from psychology and public health courses with 29.4% (Table 1).

An analysis of the teaching strategies showed that the experiences used a variety of approaches, especially group discussions (70.6%) and lectures (61.8%). Similarly, in terms of assessment strategies, there is a diversity of approaches, with a predominance of collective production (41.7%) and field diaries (37.5%). In general, IPE experiences are evaluated by students through meetings and debates (67.6%).

With regard to where IPE experiences are carried out, there is a diversity of responses, with the classroom predominating (55.9%), followed by the community and the territory (35.3%).

Finally, 50% of the experiences mapped are related to teaching in a non-compulsory subject, axis or module, and to extension activities (47.1%). It should be noted that 64.7% of the initiatives are provided for in the course’s Political Pedagogical Project (PPP); however, 11.8% of the teachers were unaware of their inclusion in the PPP and their alignment with the document guiding the pedagogical structure.

## DISCUSSION

The mapping carried out showed an overview of IPE initiatives that have been developed at USP Butantã campus, in teaching units that participated in Pet-Saúde Interprofissionalidade, which sought to strengthen IPE in undergraduate health courses. In terms of the courses involved, the number of teachers and students taking part, and the teaching and assessment strategies adopted. As such, the mapping complied with the proposal and can be applied as a diagnostic tool, helping to expand and consolidate IPE initiatives and curricular adjustments for its inclusion in all undergraduate courses offered in the health area at USP.

Among the teachers who said they participated in IPE experiences in their units, the largest proportion was associated with the Nursing, Medicine and Nutrition courses. In the literature, it is reported that Nursing is the course and subject area that leads the majority of IPE programs^(17)^, as well as the one that most closely articulates with Medicine for the development of IPE initiatives^(3)^. In the mapped scenario, it was found that teachers from the Nutrition course, followed by those from the Psychology and Public Health courses, were also developing IPE, which may indicate a characteristic of the context analyzed, with the potential to promote integration between the curricula of the various courses.

With regards to students’ participation, in addition to Medicine and Nursing, also cited in the literature as the groups where there is the more extensive engagement in interprofessional education^(3,18)^, a high level of student participation was obtained in Psychology, Physiotherapy and Nutrition. In this scenario, a wide range of students’ participation was found when compared to the literature, which is a characteristic and positive finding, since the more professionals from different professions are involved, the richer the experience becomes and the easier the curricular integration between these bachelor’s degrees will be. Analyzing the teaching contexts in which the mapped IPE experiences are inserted, it was found that they are mostly non-­compulsory, such as optional subjects and/or university extension initiatives. IPE activities are recent in undergraduate health courses in Brazil and, according to the literature, are mostly restricted to elective subjects or modules and extracurricular activities^(2,7,9,19)^. This may be related to the barriers identified for interprofessional education in undergraduate courses: weak institutional support, difficulties in finding weekly teaching periods common to the different health courses and a small number of teachers with experience of teaching interprofessional education in the various courses. However, students show interest and availability for shared learning with students from other courses when they find opportunities to do so, perhaps because they recognize the complexity of health care, the needs of users and the population and, in this sense, the relevance of interprofessionality^(10)^.

The key role of extension activities (community reach-out) in professional training should be highlighted, as it provides students with further training and teachers with continuing education, while at the same time promoting the quality of life of individuals in the surrounding communities^(20)^. Therefore, including interprofessional education in such initiatives is an effective strategy both for training students and for contextualizing interprofessional education in professional practice and in the social return that the acquisition of such skills makes possible. However, in the context analyzed, the curricularization of extension is lagging behind Resolution No. 07 of 2018 of the National Education Council (CNE in the Portuguese acronym), which establishes guidelines for extension/community reach-out in Brazilian higher education. While other institutions have made progress in the process of including extension activities in their Political Pedagogical Projects (PPP), USP is discussing the process with its units and it is a current priority for university academic management^(21)^. The optional subjects and extension activities are limited to a limited number of participants, whether students or teachers. The process of curricularizing extension can increase the number of students and teachers involved, but it will depend on how interprofessionality is effectively included as a compulsory activity for students^(21)^.

Another important aspect is the fact that more than 10% of respondents were unable to say whether or not the IPE initiative reported was included in the course’s PPP. This result reveals the need for teachers to get closer to their course’s PPP, as well as identifying points where it is possible to propose the inclusion of interprofessional education practices as curricular components^(22)^. The PPP is an instrument that includes objectives, guidelines and actions of the educational process to be developed in educational institutions, as well as characterizing both the conduct of educational practice and the evaluation criteria^(23,24)^. The lack of knowledge about whether or not the IPE initiative is included in the PPP is a relevant aspect because it may indicate a weakness in the process of institutionalizing IPE initiatives in the training activities of the analyzed courses. Therefore, ensuring and establishing the experience of interactive IPE learning in the PPP, as a way of fostering the development of skills for collaborative practice, can help overcome obstacles and bring the different professions closer together during training, increasing the possibility of achieving interprofessional and collaborative practice after training^(24)^.

The mapping showed that 45.2% of the experiences have being going on for periods between six and ten years. Thus, it is possible to conclude that, although consolidated in the institution, IPE experiences remain restricted to the non-compulsory space, being offered to a limited number of students and involving an equally scarce number of teachers, a scenario aggravated by the delay in the curricularization of extension. Although this result shows the permanence of the experiences analyzed over the years, it also reinforces the peripheral and still supporting role of interprofessional education in health^(14)^.

In order to get IPE initiatives expanded to include more students, the institution needs to align and meet its structural needs. This is because the implementation of interprofessional education in the curriculum will require greater participation by teachers, tutors and specialists, more physical space for meetings and specific spaces for practice. Therefore, in order to expand the initiatives that have already been consolidated, it has been identified that it will be essential to articulate pedagogical planning with the formation of a structure that allows IPE to be adopted.

Most IPE experiences are open-ended and can be taken at any time during the course (47.1%), while there were few reports of experiences that took place at the beginning (2.9%) or end (11.8%) of the course. The literature discusses the most effective time to include interprofessional education in initial training. Studies point out that it is recommended to be implemented on an ongoing basis, because at the beginning of the academic career it helps students understand their roles and socialize, and during the course of their training it contributes to the articulation of specific competences with collaborative competences for better health care^(3)^. In this study, IPE actions are still scattered throughout the curricula. It may be suggested that future curricular reformulations propose new IPE initiatives to ensure shared learning between students from different health courses throughout their university education.

The teaching and learning strategies most commonly adopted by teachers pointed to heterogeneous methods that fulfill very different training objectives. One study^(7)^ shows that the learning methods most adopted in IPE initiatives were interactive learning methods, such as seminar-based discussions and group problem-solving, i.e. active learning methodologies, which advocate for student autonomy and active participation in the teaching-learning process^(25)^. This result expresses the conceptual and methodological aspects of interprofessional education found in the literature, which refer to shared learning between students from two or more professions in an interactive way^(3)^. Considering the need for better planning and institutional support to assist teachers in planning interprofessional education activities, other mediation strategies could be implemented. These include simulations and case studies^(26)^, which are widely described in the literature for the positive results they achieve with students, in spite of requiring efforts and teacher integration beyond the small groups mapped out, usually of just three to four professionals, in order to be implemented effectively in student learning.

Regarding the assessment of student learning, the main ­strategies adopted were collective productions, field diaries and seminars, while methods such as written tests, portfolios and simulations had low adherence. This finding partially diverges from the literature, which encourages the adoption of methods such as portfolios and simulations^(27)^. Appropriate assessments contribute to the student’s personal and professional growth, as well as to the improvement of the educational process itself, both for the teacher and the institution, ensuring the training of professionals with the necessary attributes to carry out their future activities^(28)^. Although the methods already identified as effective and suitable for learning assessment have been less explored, it can be considered that the methods adopted fulfill the role of fitting in with active learning methodologies, as they help to articulate ideas, in the task of disseminating knowledge to the public, as well as developing research skills. The methodologies used to evaluate the experience included meetings between teachers and students and questionnaires administered to students. The literature suggests that experiences should be evaluated by involving all members in the planning and teaching of IPE, impacting over qualitative development^(29)^. Therefore, planning spaces for discussion, where everyone can express themselves and offer constructive feedback, is highly desirable when evaluating the construction of knowledge resulting from the IPE activities implemented.

In order for teachers to be able to implement teaching and assessment methodologies that are coherent and consistent with IPE, it is also necessary to move forward with proposals for teacher development. In addition to institutional support for IPE initiatives, teacher development is a key condition for strengthening teaching practice theoretically and methodologically^(30)^.

## CONCLUSION

The study presents a mapping process of the characteristics of IPE experiences in undergraduate health courses that participated in the *Pet-Saúde Interprofissionalidade* program at USP and, thus, contributes to understanding the possibilities of advancing this approach to health education in the Brazilian scenario. However, it also had limitations regarding data collection during the pandemic period, with an online survey, known to have low feedback from those invited to take part, and the length of the 33-question form.

The results show that the IPE movement in the context analyzed is incipient and involves a small number of students and teachers. Although the activities mapped are consolidated in terms of the time they are offered, they are limited to extracurricular and non-compulsory spaces. On the other hand, these IPE initiatives are powerful in supporting the implementation of new IPE practices, as well as greater coordination between courses and teaching units. For IPE to be strengthened and expanded, institutional support and teacher development are fundamental conditions.

The importance of the research lies in the diagnostic assessment which is key to advance the debate on IPE experiences within USP. The quantitative mapping of IPE experiences at USP can encourage institutional discussions on the subject, as well as inspire new experiences in other contexts. The present “portrait”, albeit provisional and situated in a certain time and space, of how IPE experiences have taken place within one of the largest universities in the country, shows a roadmap for trends that can be described in other HEIs in the future, helping with strategic planning to strengthen and expand initiatives in Brazilian universities.
